# High Return to Activity and Low Rerupture Rates Following All‐Inside Quadriceps Tendon Anterior Cruciate Ligament Reconstruction With Bone Marrow Aspirate, Demineralized Bone Matrix, and Suture Tape Augmentation

**DOI:** 10.1002/ars2.70023

**Published:** 2026-04-24

**Authors:** Jake Peterson, Claire Soucier, Jonathan Groves, Andrew L. Schaver, Timothy E. Hewett, Ethan Hahn, James Nottingham, Dana S. Lycans, Chad D. Lavender

**Affiliations:** ^1^ Joan C. Edwards School of Medicine Marshall University Huntington West Virginia U.S.A.; ^2^ Department of Orthopedic Surgery Marshall University Huntington West Virginia U.S.A.

## Abstract

**Purpose:**

To evaluate clinical outcomes of anterior cruciate ligament reconstruction (ACLR) using a quadriceps tendon autograft (QTA) with adjunctive bone marrow aspirate concentrate, demineralized bone matrix, and suture tape augmentation.

**Methods:**

A retrospective review was conducted using a prospectively maintained registry of patients who underwent all‐inside ACLR with QTA, adjunctive bone marrow aspirate concentrate, demineralized bone matrix, and suture tape augmentation between July 2018 and October 2022 at a single institution. Patients with less than a 2‐year follow‐up were excluded. A telephone survey assessed return to preinjury activity level, and patient‐reported outcomes were collected, including the International Knee Documentation Committee subjective score, ACL Return to Sport after Injury score, and Visual Analog Scale for pain. Complications such as graft rerupture and total reoperations were also recorded.

**Results:**

One hundred twenty patients (81%) received QTA ACLR, with 98 (81.7%) completing ≥2‐year follow‐up (37 female, 61 male; mean age 19.4 years, range 15‐32). Mean follow‐up was 3.4 years (range, 2.0‐5.9). All patients were cleared for full activity at 6 months postoperatively. At the time of telephone follow‐up, 90 patients (91.8%) reported they had returned to their preinjury activity levels. In all 98 patients, mean International Knee Documentation Committee and ACL Return to Sport after Injury scores were 84.0 ± 7.3 and 93.4 ± 17.2, respectively. There were 3 graft reruptures (3.1%) and 9 contralateral ACL tears (9.2%).

**Conclusions:**

All‐inside ACLR with QTA, adjunctive bone marrow aspirate concentrate, demineralized bone matrix, and suture tape augmentation showed high rates of return to preinjury activity and low graft rerupture rates at a minimum of 2 years of follow‐up.

**Level of Evidence:**

Level IV, retrospective therapeutic case series.

## Graphical abstract

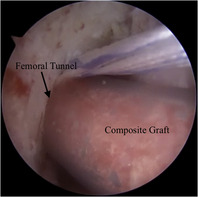

Anterior cruciate ligament reconstruction (ACLR) remains the standard treatment for restoring knee stability and enabling return to athletic activity following ACL injuries. Despite advances in surgical technique and rehabilitation, rerupture rates and secondary ACL injuries remain high.^1^, ^2^ Young athletes under the age of 25 are especially vulnerable, with secondary ACL injury rates ranging from 20% to 40%.^2^, ^3^, ^4^, ^5^ The first 2 years after ACLR represent the period of highest reinjury risk, with early return to sport (RTS) increasing the likelihood of a second injury by up to 15‐fold.^5^, ^6^ In addition to elevated reinjury rates, many patients fail to return to their preinjury level of performance, highlighting a need for improved strategies in ACLR. The biomechanical “rule of thirds” suggests that only one‐third of ACL‐injured individuals can return to full function without surgery, while another third struggle with athletic activities, and the remainder are unable to perform even daily activities.^7^ This distribution of functional outcomes is also observed in secondary injury rates, suggesting a biomechanical underpinning for graft failure and contralateral injury.

To help address these challenges, the Fertilized ACL technique has emerged, which utilizes quadriceps tendon autograft (QTA) and adds bone marrow aspirate concentrate (BMAC), demineralized bone matrix (DBM), autograft bone, and suture tape augmentation (STA) as augmentation.^8^, ^9^ These biologic enhancements are intended to promote early graft incorporation, improve bony consolidation, reduce postoperative pain, and ultimately decrease rerupture rates. BMAC contains stromal cells and growth factors, which have been shown to support bone and ACLR graft healing while modulating the inflammatory response.^10^, ^11^, ^12^, ^13^ However, STA provides mechanical support during the early phases of healing, acting as an internal brace to protect the graft.^14^, ^15^, ^16^

Early results from a small cohort using this technique have shown promising outcomes, with high return‐to‐play rates and no early graft failures.^8^ These findings suggest that the combination of biologic augmentation (BA) and STA may represent a significant advancement in ACLR, particularly for younger and high‐risk populations.

The purpose of the study was to evaluate clinical outcomes of ACLR using QTA with adjunctive BMAC, DBM, and STA. We hypothesized that ACLR with biologic and STA would result in high rates of return to preinjury activity levels and low graft rerupture rates at a minimum of 2 years postoperatively.

## METHODS

This study was approved by the Marshall University Institutional Review Board (IRB 2109731). A retrospective review of a prospectively collected registry of patients was performed. Patients who underwent ACLR with QTA, BMAC, DBM, and STA between July 2018 and October 2022 at a single institution were included. Patients were excluded if they underwent multiligament reconstructions or had less than a 2‐year follow‐up. All patients were treated by the senior author (C.D.L.). The senior author has practiced for 12 years in an academic setting after performing a sports fellowship and has Sports Certificate of Added Qualifications. Demographic information recorded included age, sex, height, weight, and body mass index (BMI). Primary outcomes were patient‐reported outcomes (PROs) (International Knee Documentation Committee [IKDC], ACL Return to Sport after Injury [ACL‐RSI], and Visual Analog Scale [VAS] pain scores) and graft reruptures at minimum 2‐year follow‐up. All patients meeting the inclusion criteria were called and surveyed at the same time irrespective of their time from surgery from surgery. Other complications were also recorded, including stiffness rates (defined as requiring manipulation under anesthesia [MUA]), contralateral knee injuries, and total reoperations. A telephone survey was conducted to obtain secondary outcomes, including if the patient was able to RTS. If the patient did not participate in sports, return to preinjury activity levels was assessed.

### Surgical Technique

**FIGURE 1:**
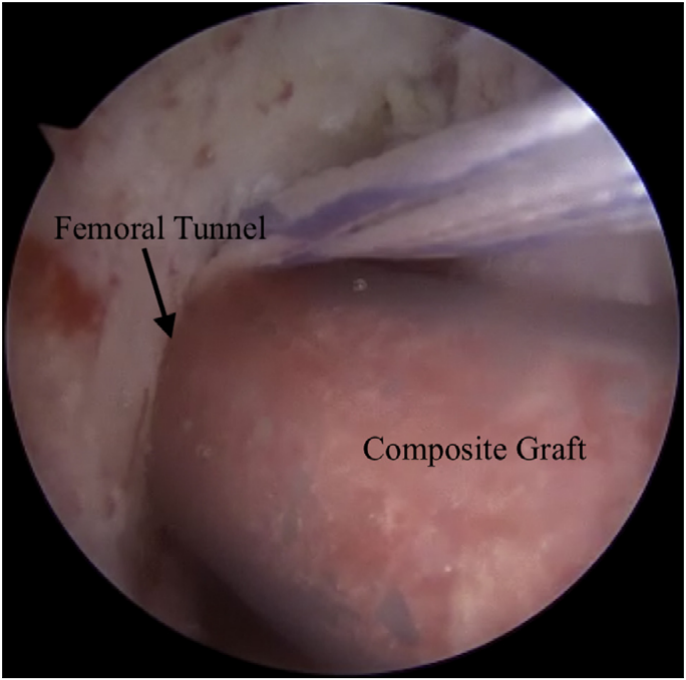
Arthroscopic view of a right knee depicting composite graft mixture injected in the femoral tunnel prior to pulling graft through tunnel.

For all procedures, ACLR with QTA, BMAC, DBM, and STA were performed as previously detailed.^9^ Indications for this technique were patients who had first‐time full‐thickness ACL tears on magnetic resonance imaging with positive Lachman examinations and wanted to return to pivoting and cutting activities. Briefly, prior to tourniquet insufflation, bone marrow was aspirated from the proximal lateral tibia (goal of 60 mL) and concentrated to 3 mL of BMAC. The tourniquet was then insufflated, and the QTA was harvested. The quad was harvested with a mini open technique taking a full‐thickness graft and the residual defect was closed with size 2 Fiberwire (Arthrex, Naples, Fl) sutures. The graft was prepared with a FiberTag attached to an RT TightRope on the femoral side and Attachable Button System (Arthex, Naples, FL) on the tibial side of the graft. The InternalBrace was passed through the femoral titanium button to run alongside the graft in reinforcement fashion. Standard diagnostic arthroscopy was performed. Meniscus repairs were performed with an all‐inside technique using a knotless repair mechanism implant. ACL tunnels were drilled with retrograde technique. While drilling the femoral tunnel, a shaver with a GraftNet (Arthrex, Naples, FL) device was used to save autograft bone. Tunnels were reamed line to line with the size of the graft. The BMAC and autograft bone were then mixed with 5 mL of DBM to create the composite graft. The mixture was injected into both femoral and tibial tunnels (Figure 1) prior to graft fixation. The graft was tensioned first at full extension, followed by the InternalBrace with the knee in full extension, secured distally with a SwiveLock Anchor (Arthex, Naples, FL).

### Postoperative Rehabilitation

Patients were all given the same postoperative protocol and allowed to bear weight as tolerated immediately after surgery. A hinged knee brace was locked in extension during ambulation for the first week after surgery. The brace was unlocked once they achieved full knee extension. If meniscus repair was performed, patients were limited to partial weight bearing for 2 weeks. Range of motion (ROM) was limited to less than 90 degrees during physical therapy. If full extension was not achieved by 6 weeks, MUA was performed, and a progressive range of motion and physical therapy program was implemented. At 6 weeks, closed chain resistance training and proprioception exercises were started. At 12 weeks, straight‐ahead running was allowed, and limb symmetry testing was initiated. Limb symmetry testing included single‐leg hop for distance, triple hop for distance, crossover hop for distance, and 6‐m timed single‐leg hop. The results for each test were averaged to give a composite percent limb symmetry. Limb symmetry testing was performed monthly starting at 3 months postoperatively until a percentage greater than or equal to 90% was achieved. All patients were allowed to RTS at 6 months postoperatively after they passed 90% limb symmetry on hop testing.

### Statistical Analysis

Descriptive statistics were performed. Continuous data were summarized using means and standard deviations while categorical data were described as frequencies and percentages. Pearson's correlation coefficient was used to determine the association between demographics (age, BMI) and outcomes (IKDC, ACL‐RSI). Characteristics of patients who experienced complications were presented separately. Statistical analysis was performed using Excel v.16.43 (Microsoft, Redmond, WA).

## RESULTS

### Demographics

Medical records from 120 ACLR procedures were reviewed. After excluding those with less than 2 years of follow‐up (n = 22), there were 98 patients (81.7%) who participated in the study. The overall mean ± standard deviation age was 19.4 ± 3.5 years (range, 15‐31 years). The cohort included 37 female and 61 male patients. The average BMI was 18.9 ± 3.8  (range, 14.1‐32.3). Mean follow‐up was 3.4 years (range, 2.0‐5.9, Table 1).

**TABLE 1:** Patient Demographics and Study Cohort

Measure	Result
Anterior cruciate ligament reconstruction procedures reviewed	120
Excluded: <2 year follow‐up	22
Final study cohort	98 patients (81.7%)
Mean age ± standard deviation (range)	19.4 ± 3.5 years (15‐31)
Mean body mass index ± standard deviation (range)	18.9 ± 3.8 (14.1‐32.3)
Mean follow‐up (range)	3.4 years (2.0‐5.9)

### Procedure Results

All patients included underwent ACLR with QTA, BMAC, DBM, and STA. Of the 98 included patients, 59% (58 patients) were found to have a meniscus tear during surgery, and 48% (47 patients) were repaired via all‐inside technique (Table 2).

**TABLE 2:** Concomitant Procedures

Procedure	Number of Patients	% of Cohort
Lateral meniscus repair	30	30.6
Medial meniscus repair	8	8.2
Medial and lateral meniscus repair	9	9.2
Partial meniscectomy	11	11.2
None	40	40.8

### Return to Activity and Patient‐Reported Outcomes

All 98 patients participated in the telephone survey and had return to activity and patient‐reported outcomes available. Overall, the average time of telephone follow‐up was 3.4 years after surgery (range, 2.0‐5.9). All patients were cleared for full activity at 6 months. At the time of telephone follow‐up, 90 patients (91.8%; 33 females, 57 males) reported that they had returned to their preinjury activity levels. In all 98 patients, mean IKDC and ACL‐RSI scores were 84.0 ± 7.3 and 93.4 ± 17.2, respectively. For male patients, mean IKDC was 84.4 ± 5.9 and ACL‐RSI was 93.7 ± 16.2. For female patients, mean IKDC was 83.2 ± 9.3 and ACL‐RSI was 92.8 ± 19.0. Mean VAS pain scores were <1 for all patients (0.38 ± 1.1 points, Table 3). Age and BMI did not significantly correlate with mean IKDC (Age: *r* = −0.16, *P* = .86; BMI: *r* = −0.12, *P* = .79) or ACL‐RSI (Age: *r* = −0.13, *P* = .81; BMI: *r* = −0.08, *P* = .85).

**TABLE 3:** Telephone Survey and Patient‐Reported Outcomes

Measure	Result
Final cohort	98
Average final follow‐up	3.4 years (Range 2.0‐5.9)
Cleared for full activity	100% (at 6 months)
Returned to preinjury activity level	90 patients (91.8%)
IKDC	84.0 ± 7.3
ACL‐RSI	93.4 ± 17.2
VAS pain score	0.38 ± 1.1 (all <1)

ACL‐RSI, Anterior Cruciate Ligament Return to Sport after Injury; IKDC, International Knee Documentation Committee; VAS, visual analog score.

There were 8 patients (4 male, 4 female) who reported the inability to return to full activity after surgery. The mean age of these patients was 18.5 ± 2.0 years. Two patients sustained rerupture and underwent revision ACLR. The remaining 6 patients chose not to RTS for personal reasons. Mean IKDC and ACL‐RSI scores at the time of telephone follow‐up were 70.0 ± 16.8 and 80.5 ± 19.3, respectively.

### Complications

Three female patients did not achieve full extension by 6 months and underwent MUA at mean 57 days postoperatively. Of these patients, 1 patient sustained rerupture, and the remaining 2 patients were able to return to full activity. Overall, there were 3 graft reruptures (3.1%), including a high‐school female soccer player who sustained a repeat pivot‐shift injury 14 months after surgery and a high‐school male track athlete who tripped on a hurdle 8 months after surgery. This patient opted not to undergo revision ACLR. Details of the third rerupture were not available, as the patient underwent repeat surgery elsewhere. In addition, 9 contralateral ACL tears (9.2%) occurred at mean 36 months after surgery. Four patients underwent reoperations (4.1%), including 2 revision ACLR, 1 medial meniscus tear, and 1 second look for persistent knee pain, without pathology noted.

## DISCUSSION

The findings of this study suggest that ACLR with QTA, BA (BMAC and DBM), and STA is a safe and effective technique, with high rates of return to preinjury activity levels and low graft failure rates at a minimum of 2 years of follow‐up. In this cohort of young, active patients, 91.8% returned to their preinjury level of activity, and only 3.1% sustained graft reruptures. These findings confirm our hypothesis. Secondary outcomes were also encouraging; mean IKDC and ACL‐RSI scores were 84.0 and 93.4, respectively, indicating strong functional recovery and psychological readiness to RTS. Additionally, the reoperation rate was low at 4.1%, and reported pain levels were minimal (mean VAS score <1).

Reinjury remains one of the most concerning complications following ACLR, particularly in young athletes. The rerupture rate of 3.1% in this study is markedly lower than reported in similar populations and may reflect the protective role of both BA and STA. Prior studies have suggested that BMAC and DBM can enhance tendon‐to‐bone healing by promoting osteogenesis and reducing inflammation.^12^, ^17^, ^18^ Additionally, STA helps provide mechanical support during early graft healing, and this has been associated with improved knee stability in preclinical models.^14^, ^19^ The combination of biologic and mechanical reinforcement in this study may have contributed to earlier RTS clearance and fewer graft failures.

RTS testing in this study was achieved between 3 and 6 months postoperatively, with all patients meeting ≥90% limb symmetry index prior to clearance. The ability to meet functional milestones early without compromising outcomes is particularly relevant in young athletic populations, where pressure to return to competition can be significant.^20^, ^21^ Despite early RTS, rerupture rates remained low in our study, which contrasts with prior findings suggesting increased risk of reinjury with RTS before 12 months.^1^, ^22^ This raises the possibility that the biologic and mechanical enhancements used in this technique may allow for accelerated yet safe rehabilitation pathways.

The mean IKDC and ACL‐RSI scores observed (84.0 and 93.4, respectively) are consistent with high functional recovery and psychological readiness to RTS. Previous studies have shown that ACL‐RSI scores greater than 60 are predictive of successful RTS.^23^, ^24^, ^25^ The high ACL‐RSI values reported in this study further support the efficacy of this technique in addressing not only physical but also psychological components of recovery. One disadvantage of BA is cost added to the procedure. The cost is variable but can range from $1,000 to $2,000 per procedure.

The complication rate in this series was low. Only 3 patients (3.1%) required MUA for postoperative stiffness, and all but 1 eventually returned to full activity. Notably, the contralateral ACL tear rate (9.2%) is similar to rates reported in other high‐risk populations and underscores the importance of bilateral neuromuscular rehabilitation in young athletes.

### Limitations

This study has several limitations. First, it was retrospective in nature and lacked a control group, limiting the ability to make direct comparisons with traditional ACLR techniques. Second, patient‐reported outcomes were collected via telephone survey, which may introduce response and recall bias. Additionally, important demographic data, including time to surgery, right/left knee operated, sport (no sport/contact/no contact sport), and cause of injury, were not collected. Furthermore, the Minimal Clinically Important Difference has not been established for the procedure studied, so we were unable to include that information. Lastly, while the follow‐up was a minimum of 2 years, longer‐term studies are needed to assess the durability of outcomes and the potential protective effect of BA over time. Further prospective, randomized studies are warranted to confirm these findings and determine the relative contributions of each component of the biologically augmented reconstruction.

## CONCLUSIONS

All‐inside ACLR with QTA, BMAC, DBM, and STA showed high rates of return to preinjury activity and low‐graft rerupture rates at a minimum of 2 years of follow‐up.

## DISCLOSURES

The authors (T.E.H., C.D.L.) declare the following financial interests/personal relationships, which may be considered as potential competing interests: T.E.H. reports a relationship with Sparta Science that includes: consulting or advisory. C.D.L. reports a relationship with Arthrex Inc that includes: consulting or advisory, funding grants, paid expert testimony, and speaking and lecture fees. The other authors (J.P., C.S., J.G., A.L.S., E.H., J.N., D.S.L.) declare that they have no known competing financial interests or personal relationships that could have appeared to influence the work reported in this paper.
